# Hyperammonemic encephalopathy in a patient receiving fluorouracil/oxaliplatin chemotherapy

**DOI:** 10.1002/ccr3.1422

**Published:** 2018-02-13

**Authors:** Hiromi Ihoriya, Hirotsugu Yamamoto, Taihei Yamada, Kohei Tsukahara, Kanae Inoue, Tetsuya Yumoto, Hiromichi Naito, Atsunori Nakao

**Affiliations:** ^1^ Department of Emergency and Critical Care Medicine Okayama University Graduate School of Medicine Dentistry and Pharmaceutical Sciences 2‐5‐1 Shikata‐cho, Kita‐ku Okayama‐shi Okayama 700‐8558 Japan

**Keywords:** Chemotherapy, colon cancer, fluorouracil, hyperammonemia, mental disorder

## Abstract

Hyperammonemia is a rare adverse effect of 5‐fluorouracil (5‐FU) therapy, but can be very serious, even fatal. Physicians must be aware that hyperammonemic encephalopathy sometimes develops as an adverse event after 5‐FU therapy.

## Introduction

Systemic chemotherapy with 5‐fluorouracil (5‐FU) combined with other agents, including folinic acid, is an accepted therapy for recurrent or unresectable colorectal cancer. Even though hyperammonemia is known to be an adverse side effect of 5‐FU, hyperammonemia‐induced disturbance of consciousness is an unusual finding 1, 2. We report a hyperammonemia case with normal liver function receiving chemotherapy and exhibiting an altered mental status in the emergency department. Acute hyperammonemia, defined as elevated plasma ammonia, is a medical emergency; immediate action must be taken to curtail permanent brain damage.

Ammonia is normally detoxified in the liver and extrahepatic tissues by conversion into urea and glutamine, respectively. In the brain, glutamine synthesis is largely confined to astrocytes, and excess glutamine compromises astrocyte morphology and function in hyperammonemia. Ammonia accumulation in the brain causes cerebral altered mental status, edema, coma, seizures, and death 3, 4.

Although severely abnormal liver function is the most common cause of hyperammonemia, liver disease is absent in some cases. Physicians should be aware that administration of 5‐fluorouracil is one of the significant causes of hyperammonemia.

## Case Report

A 64‐year‐old male was transferred to our emergency department due to incontinence and impaired consciousness. His airway was patent, but his Glasgow Coma Scale at the time of the presentation was deteriorated (E3V2M4). He had no history of diabetes or hypertension, did not drink alcohol regularly, and had ceased smoking several years prior. The patient was not constipated before admission. His wife stated that he played golf the day before visit, and he presented dry mouth and felt thirsty the night before admission. He had undergone left hemicolectomy for pT3N1M0 stage IIIa colon cancer 3 years earlier. His postoperative course was uneventful and was subsequently given 5‐FU/folinic acid and oxaliplatin at doses of 1.5 g/m^2^, 75 mg/m^2^, and 85 mg/m^2^, respectively.

Arterial blood gas levels were abnormal: pH, 7.432; pCO_2_, 27 Torr; pO_2_, 127.0 Torr; glucose, 172 mg/dL; base excess −5.5, indicating lactic acidosis with respiratory compensation. His lactic acid level was 5.1 mmol/L (0.44–1.78 mmol/L). An emergent serum biochemistry evaluation indicated normal liver and kidney function: total bilirubin, 0.53 mg/dL; aspartate aminotransferase, 19 IU/L; alanine aminotransferase, 6 IU/L; blood urea nitrogen, 10.0 mg/dL; and creatinine, 0.76 mg/dL. His serum ammonia level was 338 *μ*g/dL (30–86 *μ*g/dL). Serum sodium and potassium levels were of 151.1 and 4.8 mEq/L, respectively. Computed tomography did not reveal any intracranial pathology such as hematoma, stroke, or metastasis. Magnetic resonance imaging revealed lacunar infarctions close to the cerebral ventricle. However, we did not observe any symptoms associated with these lesions. The patient was diagnosed with disturbance of consciousness caused by hyperammonemia. We stopped chemotherapy and started intravenous glycerol infusion, diuretics, and fluid replacement. The patient became fully conscious with a serum ammonia level of 31 *μ*g/dL by the next day.

## Discussion

The frequency of hyperammonemia induced by 5‐FU‐based therapy has been reported as 5.7% (16/280) among cancer cases treated with 24‐h 5‐FU infusion (2600 mg/m^2^/week) and leucovorin (300 mg/m^2^/week) 5. The actual mechanisms of hyperammonemia caused by 5‐FU administration are unknown. Possibly, accumulation of fluorocitrate, a by‐product of 5‐FU metabolism, restricts the Krebs cycle, causing impairment of the adenosine triphosphate‐dependent urea cycle. Accordingly, ammonia isn't converted to urea, which consequently leads to ammonia accumulation. Weight loss with muscle atrophy is also considered an important factor impacting the catabolism of ammonia.

Basically, hyperammonemia develops when ammonia is either over‐generated or inadequately cleared from the serum with the urea cycle. The accumulated ammonia is metabolized into glutamine, which has been suggested to increase cerebral edema and intracranial pressure. Because extrahepatic tissues do not contain a functioning urea cycle, ammonia is detoxified in these tissues as glutamine which is then released to the circulation to eventually reach the liver as a source of ammonia for urea synthesis. Muscle occupies such a large volume it is a major sink for whole body ammonia, presumably trapped as glutamine. This sink will be disrupted in cachexic hyperammonemic patients 6.

Hyperammonemic encephalopathy usually occurs in patients with serious liver dysfunction. However, certain disorders without obvious liver disease might be associated with hyperammonemia, including inherited urea cycle enzyme deficiencies, hematologic malignancy, and Reye's syndrome 3, 7. Manifestations of hyperammonemia include aggression, nocturnal delirium, disorientation, delusions, loss of memory, restlessness, coma, and convulsive seizures; brain edema can cause death. Sugar and alcohol consumption, infection, medication, and/or surgery often induce symptoms.

Since kidney dysfunction is a risk factor for 5‐FU‐induced hyperammonemia, close attention needs to be paid to patients with renal dysfunction treated with 5‐FU‐based chemotherapy. It has long been known the breakdown of a portion of the urea generated in the body by intestinal bacteria contributes to the high level of ammonia in normally found in the portal vein 8. Ongoing constipation may lead to excessive ammonia generation, since most ammonia is produced in the colon through the action of amino acid oxidase and bacterial urease 9. Our patient did not present with constipation; however, dehydration due to sweating might have been responsible for hyperammonemia in our patient 10. The present case did not assess the metabolic abnormality, since an episode of 5‐FU administration existed. We may doubt metabolic abnormality in cases where the cause of hyperammonemia is unknown without a history of hepatic disease.

Treatment should be started as soon as hyperammonemic encephalopathy is suspected, since clinical prognosis is highly related to early diagnosis and treatment. In addition to discontinuation of the medication inducing hyperammonemia, essential amino acid supplementation, protein restriction, and adequate hydration are required. Valproic acid and 5‐FU level should be discontinued. Pharmacists, physicians, and nurses can play a significant role in educating patients receiving 5‐FU about possible side effects and when they should go to the emergency room.

## Conclusion

Hyperammonemia is a rare adverse effect of 5‐FU therapy, but can be very serious, even fatal. Greater recognition of the syndrome by monitoring plasma ammonium levels in patients with neurological symptoms is imperative, leading to immediate diagnosis and swift administration of therapy. Physicians must be aware that hyperammonemic encephalopathy sometimes develops as an adverse event after 5‐FU therapy.

## Informed Consent

Consents, permissions, and releases were obtained where authors wished to include case details or images of patients and any other individuals in publication.

## Authorship

HI, HY, TY, KT, KI, TY, and HN: participated in patient management and data collection, contributed to the interpretation of the case, and critically reviewed the manuscript. AN: participated in patient management, collected and analyzed the data, wrote the manuscript, and critically reviewed the manuscript. All authors approved the final manuscript as submitted.

## Conflict of Interest

None declared.
